# Left paraduodenal hernia: a rare cause of recurrent abdominal pain: case report

**DOI:** 10.11604/pamj.2021.40.135.32065

**Published:** 2021-11-03

**Authors:** Leena Hatem Moshref, Shumukh Hassan Alqahtani, Zaid Abdulrahim Majeed, Jameel Miro

**Affiliations:** 1 Doctor Soliman Fakeeh Hospital, Jeddah, Saudi Arabia,; 2 Umm Al-Qura University, Mecca, Saudi Arabia

**Keywords:** Paraduodenal hernia (PDH), laparoscopy, case report

## Abstract

Internal hernias are quite rare, accounting for fewer than 1% of all abdominal hernias. Moreover, the most frequent cause of internal herniation is paraduodenal hernia (PDH). Diagnosing paraduodenal hernias can be difficult due to the wide range of symptoms that can occur. It is a case report of paraduodenal hernia that was diagnosed and managed in a tertiary center. We describe the case of a 55-year-old male patient who had been experiencing nonspecific abdominal discomfort for the last 5 years and had several comorbidities and positive serology. An abdominal computed tomography (CT) revealed that he had a left PDH, which was effectively treated with laparoscopic surgical repair. The study's strength is that it was correctly identified by CT and subsequently laparoscopically corrected. A gastrografin swallow study was also performed postoperatively to ensure there was no leak. The study's flaw is that the patient was misdiagnosed for five years in other medical facilities. Because paraduodenal hernia is an uncommon diagnosis, it's important to keep it in mind as one of the differential diagnoses for persistent discomfort. It also needs accurate imaging in order to identify and successfully manage it. To demonstrate the relevance of this uncommon condition, future research is needed to report on any misdiagnosis or treatment. To conclude, nowadays the best option for paraduodenal hernia repair is laparoscopic surgery. However, because it can present with a wide variety of symptoms and diagnosis might be challenging, it demands a high index of suspicion. The gold standard for identifying paraduodenal hernia is still a CT scan of the abdomen. Patients who are stable and have no signs of intestinal obstruction might decide to have their hernia repaired to avoid future problems. To demonstrate the relevance of this uncommon condition, future research is needed to report on any misdiagnosis or treatment.

## Introduction

Internal abdominal hernias are produced by the protrusion of abdominal viscera through a defect in the peritoneum or mesentery, which can be congenital or acquired [1]. Internal hernia is also an uncommon condition, accounting for less than 1% of all abdominal hernias [1]. Internal hernia is divided into six types: paraduodenal hernia (PDH), pericecal, transmesenteric, followed by hernia of the foramen of Winslow, intersigmoid, and paravesical hernia with PDH being the most common congenital hernia [1]. PDH can be categorized as either right-sided (25%) or left-sided (75%) based on radiological findings [1]. PDH might be asymptomatic or present with a variety of symptoms that make it difficult to diagnose, such as, chronic abdominal pain, and bowel obstruction, can lead to intestinal perforation if not detected and treated surgically [2].

We present a 55-year-old man with multiple comorbidities and a positive serology. A left PDH was identified with abdominal CT and successfully treated with laparoscopic repair and favorable clinical outcomes. Our case was unique because the patient had multiple comorbidities, chronic abdominal pain and corrected identified by imaging. A gastrografin swallow study was also performed postoperatively to ensure there was no leak.

## Patient and observation

**Patient information:** a 55-year-old man, known case of coronary artery disease, HCV, hyperuricemia, and dyslipidemia. On February 1^st^, 2021, he went to the surgical clinic with a 5-year history of persistent abdominal discomfort in the left upper quadrant. The ache was dull and came and went in a random manner. However, the discomfort has been worse in the previous two months. It was linked to nausea and diarrhea. Right coronary artery stent, coronary artery bypass graft (CABG), open right inguinal hernia repair, left laparoscopic inguinal hernia repair, and appendectomy are among his previous procedures. He was taking clopidogrel, rosuvastatin, allopurinol, and bisoprolol. Systemic review was unremarkable. Family and psychosocial history were unremarkable.

**Clinical findings:** examination revealed that vital signs were within normal limits, that the patient was alert, conscious, and oriented, that the abdomen was soft and lax, and there was no tenderness or guarding. Laboratory tests showed that complete blood count (CBC) and coagulation profile were both normal. Serology revealed HCV antibodies 110, as well as normal HIV, HBV.

**Timeline:** the patient arrived at the surgery clinic on February 1^st^, 2021. Both the history and examination were completed. The next day, laboratory tests revealed normal findings, with the exception of +ve HCV antibodies. A colonoscopy was performed on February 4^th^, 2021, and the results were normal. An abdominal CT on February 8^th^, 2021, confirmed a left paraduodenal hernia. The patient was taken to the hospital for surgical treatment of the paraduodenal hernia after a week. His paraduodenal hernia was repaired laparoscopically. A small bowel barium follow-up was conducted on the first postoperative day (February 17^th^, 2021), and it was normal. The patient was released home in a stable condition on February 18^th^, 2021. The patient was seen at the surgical clinic 10 days after being discharged (February 27^th^, 2021), and the appointment was unremarkable.

**Diagnostic assessment:** on February 4^th^, 2021, he underwent a colonoscopy, which came out normal. On February 8^th^, 2021, an abdomen CT scan was performed to confirm the diagnosis of a left paraduodenal hernia. The diagnosis was challenging as the patient had been having chronic abdominal pain for 5 years and was misdiagnosed in other healthcare facilities. A left paraduodenal hernia was determined as the final diagnosis.

**Figure 1:**
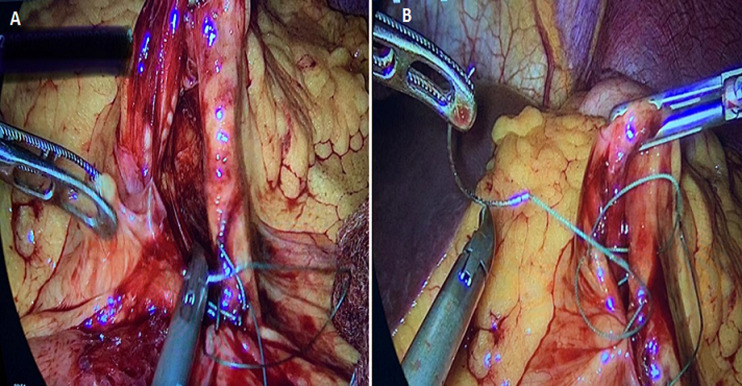
closure of left paraduodenal hernia using running 2-0 Ethibond suture

**Figure 2:**
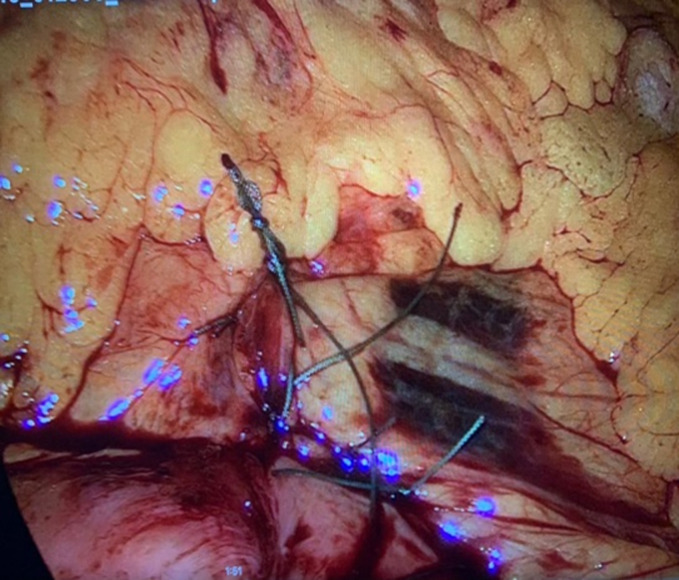
closure of ligament of Treitz by 2 interrupted sutures

**Therapeutic intervention:** on February 16^th^, 2021, the patient was hospitalized for elective hernia repair. Clopidogrel was stopped five days before surgery. Prior to surgery, he was given 1500 mg of cefuroxime. The patient was draped, prepared, and positioned supine. The port was inserted into the supraumbilical space using the Hasson method and an 11 mm trocar. For pneumoperitoneum, a 14-mmHg pressure flow was utilized. Three 5 mm trocars were all positioned in the same way. The patient was kept in a reversed Trendelenburg posture, and all of the small bowel was spontaneously relocated into the right iliac fossa. Between the inferior mesenteric vein and the ligament of Treitz, the left paraduodenal hernia orifice was visible. The Treitz ligament was discovered, and the whole intestine was examined until it reached the ileocecal junction. The remainder of the mesocolon remained intact, and there was no right paraduodenal opening. The stomach, liver, and colon were all normal. The left paraduodenal hernia was closed with a running 2-0 Ethibond suture (Figure 1). To attach the Treitz ligament to the defect, two additional interrupted sutures were utilized (Figure 2). Under direct vision, hemostasis was maintained, and trocars were removed. The fascia was closed with vicryl, while the skin was closed with monocryl. The dressing was placed on the wound. The patient responded well to the surgery and was extubated before being moved to the post-anesthesia care unit and then to the normal surgical ward.

The patient was given ringer lactate, analgesia (regular paracetamol 1 g QID IV, tramadol PRN 100 mg TID IV), hyoscine 10 mg TID oral, metoclopramide PRN 10 mg TID IV and enoxaparin 40 mg once daily SC (beginning 6 hours after surgery) as well as his regular home medication.

**Follow-up and outcome of interventions:** the patient stayed two days in the hospital postoperatively. On the first post-operative day, the patient was doing well and complaining of mild abdominal pain in the lower quadrant. He tolerated a liquid diet well, with no nausea, vomiting and passed bowel movements. His vital signs were normal, he was alert, conscious, and oriented, his abdomen was soft and lax, and the dressing was dry and clean upon examination. On February 17^th^, 2021, he underwent a small bowel barium follow-up, which came back normal. The patient felt much better on the second postoperative day, and his discomfort had gone, so he was sent home. The patient was advised to follow a liquid diet for one week and to take paracetamol and tramadol when needed for pain.

The patient was examined at the surgical clinic one week after being discharged on February 27^th^, 2021. He was fine and didn't have any concerns. The examination was unremarkable. There were no adverse and unanticipated events.

## Discussion

Paraduodenal hernias are a rare congenital hernia that arises when abdominal contents become stuck in the colon's mesentery, obstructing the small intestine [1]. The exact process of paraduodenal hernias is unknown, and there are a variety of ideas and speculations about where they come from. The most widely acknowledged reason for intestinal hernias is a rotation and fixation error in the gut [3]. The complete rotation of the proximal loop of the midgut, the duodenum, and the jejunum is thought to be the etiology of paraduodenal hernia. As we get older, the remains of the proximal end and midgut move into the same compartment under the stomach, as well as an unfixed portion of the descending colon's mesocolon. The rest of the proximal end and midgut migrate into the same compartment under the stomach, together with an unfixed section of the descending colon's mesocolon. As a result, the compartment's anterior wall is made up of the stomach, mesocolon of the distal transverse colon, and descending colon. The inferior mesenteric artery and vein, which also borders the gap through which the terminal ileum emerges to join the caecum, constitute the medial edge of the hernial sac [4]. Paraduodenal hernia accounts for fewer than 1% of all intestinal obstruction cases, although it can account for up to 5.8% of all small intestine obstruction cases [5]. Internal hernias have a three-to-one male/female sex ratio [6]. As in our study, the patient was a male. Asymptomatic paraduodenal hernias are discovered by chance during a laparotomy or autopsy [4] In symptomatic people, clinical symptoms might vary from regular bouts of stomach pain to severe intestinal blockage or strangulation [4]. Our patient had been suffering from severe stomach discomfort for the past five years and had been misdiagnosed.

In 1921, Kummer published the first case report on the use of barium swallow to diagnose PDH prior to surgery [7]. Modern imaging techniques such as CT have made it simpler to detect PDH preoperatively, which had previously been challenging due to its wide range of symptoms [8]. An abdominal CT scan is the gold standard for diagnosing PDH. The left PDH is distinguished by a cluster of bowel loops above or at the same level as the Treitz ligament, causing a small mass effect on the stomach's posterior wall. Anomalies of the mesenteric vascular system have also been reported [9]. Because these types of hernias have a 50 percent lifetime probability of complications, such as strangling and intestinal obstruction, which have a mortality rate of 20-50 percent [9], surgical surgery is suggested once the hernia is found. In addition, surgical therapy for any kind of hernia entails decreasing herniated contents and restoring normal anatomical structure by repairing the hernial orifice, as well as excision of necrotic portions if necessary [9,10]. Furthermore, the left colic artery or inferior mesenteric arteries must be identified to avoid damage in the left PDH [10].

The study's strength is that it was correctly identified by CT and subsequently laparoscopically corrected. Also, a patient with several comorbidities might benefit from a laparoscopic approach. A small bowel barium follow-through can be performed postoperatively to ensure there was no leak. The study's flaw is that the patient was misdiagnosed for five years in other medical facilities.

Our limitation is that patient had several comorbidities, positive serology, and was taking oral anticoagulants, making it difficult to make a management decision. The decision to do a laparoscopy was difficult, but it was completed managed successfully. This might influence future study into the use of a laparoscopic technique in a patient with multiple morbidities and a positive serology.

## Conclusion

Nowadays the best option for paraduodenal hernia repair is laparoscopic surgery. However, because it can present with a wide variety of symptoms and diagnosis might be challenging, it demands a high index of suspicion. The gold standard for identifying paraduodenal hernia is still a CT scan of the abdomen. Patients who are stable and have no signs of intestinal obstruction might decide to have their hernia repaired to avoid future problems. To demonstrate the relevance of this uncommon condition, future research is needed to report on any misdiagnosis or treatment.

## References

[1] Ghahremani GG (1984). Internal abdominal hernias. Surg Clin North Am.

[2] Bartlett MK, Wang C, Williams WH (1968). The surgical management of paraduodenal hernia. Ann Surg.

[3] Shinohara T, Okugawa K, Furuta C (2004). Volvulus of the small intestine caused by right paraduodenal hernia: a case report. J Pediatr Surg.

[4] Maudar KK, Gaur RK (1994). Paraduodenal hernia (a case report). Med J Armed Forces India.

[5] Hassani KI, Aggouri Y, Laalim SA, Toughrai I, Mazaz K (2014). Left paraduodenal hernia: a rare cause of acute abdomen. Pan African Medical Journal.

[6] Fan HP, Yang AD, Chang YJ, Juan CW, Wu HP (2008). Clinical spectrum of internal hernia: a surgical emergency. Surg Today.

[7] Nuño-Guzmán CM, Arróniz-Jáuregui J, Hernández-González C, Reyes-Macías F, Nava-Garibaldi R, Guerrero-Díaz F (2011). Right paraduodenal hernia in an adult patient: diagnostic approach and surgical management. Case Rep Gastroenterol.

[8] Parmar BP, Parmar RS (2010). Laparoscopic management of left paraduodenal hernia. J Minim Access Surg.

[9] Al-Khyatt W, Aggarwal S, Birchall J, Rowlands TE (2013). Acute intestinal obstruction secondary to left paraduodenal hernia: a case report and literature review. World J Emerg Surg.

[10] Falk GA, Yurcisin BJ, Sell HS (2010). Left paraduodenal hernia: case report and review of the literature. BMJ Case Rep.

